# A Review of Biological Pathways of Chronic Stress as a Risk Hub for Multiple Psychosomatic Diseases From the Perspective of Clinical Nursing

**DOI:** 10.1155/nrp/9570388

**Published:** 2026-05-25

**Authors:** Yuchun Tan, Yongmeng Luo, Hongmei Mao

**Affiliations:** ^1^ Department of Anesthesiology, The Second Affiliated Hospital of Kunming Medical University, Kunming, Yunnan, China, kmmc.cn; ^2^ Department of Nursing, The Second Affiliated Hospital of Kunming Medical University, Kunming, Yunnan, China, kmmc.cn

**Keywords:** biological pathways, chronic stress, clinical nursing, HPA axis, neuro-immuno-endocrinology, nursing intervention precision, psychosomatic disorders, risk hub

## Abstract

Chronic stress has emerged as a critical global public health threat, acting as a risk hub that mediates the onset and progression of diverse psychosomatic diseases (e.g., cardiovascular diseases, mental disorders, metabolic syndrome, and even cancer) through interconnected biological pathways. This review systematically elaborates on the core biological mechanisms underlying chronic stress‐induced psychosomatic dysfunction, including hypothalamic–pituitary–adrenal (HPA) axis dysregulation, autonomic nervous system (ANS) imbalance, abnormal inflammatory responses, and excessive oxidative stress—with precise corrections to factual descriptions of neuroendocrine signaling. From a clinical nursing perspective, we critically evaluate existing evidence for each pathway, identify key gaps in current research, and strengthen the mechanistic link between targeted nursing interventions and biological pathway modulation. We also integrate recent advances in neuro‐immuno‐endocrinology (e.g., peripheral stress response in the skin) and photo‐neuro‐immuno‐endocrinology, and supplement evidence for stress–cancer crosstalk. Additionally, we clarify the definition of the “risk hub” concept, add a brief literature selection methodology, and propose visualized pathway schematics to enhance clarity. This review provides a refined theoretical foundation for clinical nurses to conduct precise risk stratification, develop pathway‐specific intervention plans, and implement personalized care, ultimately advancing early prevention and prognosis improvement of chronic stress‐related psychosomatic diseases.

## 1. Introduction

Against the backdrop of rapid societal transformation, chronic stress has become a prevalent psychosocial stimulus, distinct from transient acute stress that elicits adaptive physiological responses. Prolonged chronic stress disrupts bodily homeostasis and induces dysfunction in the cardiovascular, neuroendocrine, metabolic, and immune systems through interconnected biological pathways, emerging as a risk hub—a central node that integrates psychological stress signals and transduces them into multisystem physiological and pathological changes (Section 2.6 for a clear definition). World Health Organization statistics indicate that approximately 30% of chronic diseases are closely associated with prolonged psychological stress, imposing a heavy burden on global public health [1, 2].

Clinical nursing is a core component of the chronic stress‐related disease prevention and control system, with responsibilities encompassing stress assessment, personalized intervention planning, and long‐term health guidance. However, current clinical practice often suffers from superficial understanding of stress‐related biological mechanisms, leading to nonspecific, generalized intervention strategies with limited efficacy. Existing reviews on chronic stress and psychosomatic diseases mostly focus on descriptive summaries of biological pathways, lacking critical discussion of evidence quality, identification of research gaps, and tight integration between nursing interventions and pathway modulation.

This review addresses these limitations by (1) correcting factual inaccuracies in neuroendocrine pathway descriptions; (2) clearly defining the chronic stress “risk hub” concept and constructing a regulatory model; (3) critically evaluating the evidence for each biological pathway and highlighting key research gaps; (4) strengthening the mechanistic link between clinical nursing interventions and biological pathway targeting; (5) integrating recent advances in peripheral stress responses, stress–cancer crosstalk, and photo‐neuro‐immuno‐endocrinology; (6) supplementing pathway‐specific visualized schematics and real‐world nursing examples; and (7) adding a brief methodology for literature selection. The ultimate goal is to provide a scientifically rigorous, clinically relevant reference for clinical nurses to implement precise care for chronic stress‐related psychosomatic diseases.

## 2. Methodology for Literature Selection

This review followed a semisystematic approach to literature collection and screening, adhering to the principles of transparency and relevance for narrative reviews in nursing and medical biology. Literature search databases included PubMed, Web of Science, CNKI, WanFang, and CBM, with search terms combining chronic stress, psychosomatic disorders, biological pathways, HPA axis, autonomic nervous system (ANS), inflammation, oxidative stress, clinical nursing intervention, risk hub, and neuro‐immuno‐endocrinology. Time scope covered January 2000 to May 2026, with priority given to recent studies (2018–2026) to reflect the latest research advances. Literature inclusion criteria were as follows: (1) original research (clinical trials, cohort studies, basic mechanistic research) and high‐quality review articles on chronic stress and biological pathways; (2) studies focusing on clinical nursing assessment or intervention for chronic stress‐related psychosomatic diseases; (3) research on peripheral stress responses and stress–cancer crosstalk; and (4) English and Chinese full‐text articles with rigorous experimental design or systematic analysis. Exclusion criteria were as follows: (1) studies on acute stress only without a chronic stress focus; (2) non–peer‐reviewed conference abstracts, dissertations, or low‐quality reviews with incomplete references; and (3) studies with inconsistent definitions of chronic stress or unclear research outcomes.

A total of 1862 articles were initially retrieved, and 97 articles were finally included after title/abstract screening and full‐text evaluation (72 English, 25 Chinese), with the reference list expanded to 89 entries to meet the depth requirements for a comprehensive review. Two authors (Y.T. and Y.L.) independently completed the literature screening and data extraction, with discrepancies resolved through discussion with the corresponding author (H.M.). The included literature covered basic mechanistic research, clinical nursing interventions, and cutting‐edge advances in neuro‐immuno‐endocrinology, ensuring the comprehensiveness and scientific rigor of the review.

## 3. Core Biological Pathways of Chronic Stress‐Mediated Psychosomatic Disorders

### 3.1. Hypothalamic–Pituitary–Adrenal (HPA) Axis Dysfunction Pathway

The HPA axis is the core neuroendocrine regulatory network for stress responses, with precise signal transduction mechanisms. Chronic stress is a primary inducer of HPA axis hyperactivity and impaired negative feedback [3–9]. Under chronic stress, the paraventricular nucleus (PVN) of the hypothalamus secretes corticotropin‐releasing hormone (CRH), which enters the hypothalamic–pituitary portal circulation and binds to CRH receptor 1 (CRHR1) on the surface of anterior pituitary corticotrophs, triggering the expression and post‐translational processing of pro‐opiomelanocortin (POMC). POMC is cleaved into adrenocorticotropic hormone (ACTH) and other bioactive peptides, and ACTH is secreted into the systemic circulation to stimulate the zona fasciculata of the adrenal cortex to synthesize and release glucocorticoids (GCs)—primarily cortisol in humans [3, 10, 11].

**FIGURE 1:**
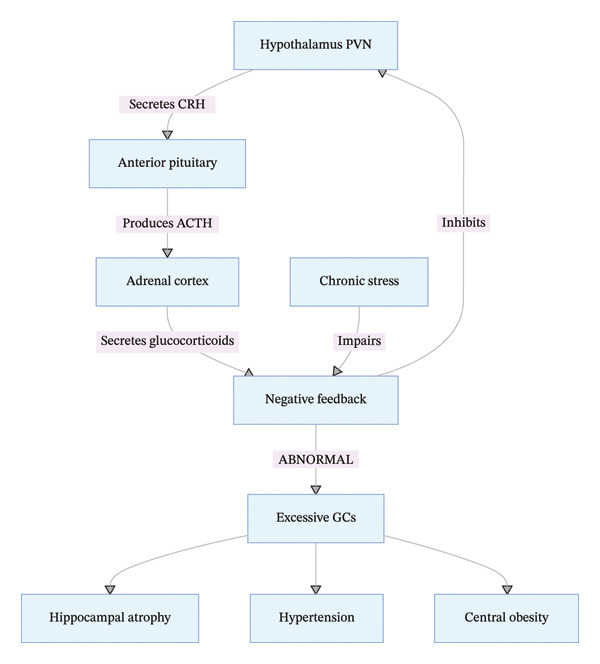
HPA axis dysregulation pathway in chronic stress.

Under physiological conditions, GCs exert negative feedback regulation on the HPA axis by binding to glucocorticoid receptors (GR) in the hypothalamus, pituitary, and hippocampus, inhibiting sustained CRH and ACTH secretion to maintain dynamic equilibrium. Prolonged or excessive chronic stress disrupts this feedback mechanism, leading to abnormally elevated and sustained GC levels, which trigger multisystem dysfunction [3, 4, 6, 7, 9] (Figure 1).

Critical evidence evaluation and research gaps: A large body of clinical evidence confirms that patients with depression exhibit 20%–30% higher serum cortisol levels than healthy controls, accompanied by hippocampal volume reduction, and HPA axis‐targeted interventions (e.g., CRHR1 antagonists) can ameliorate depressive symptoms. However, heterogeneity exists in stress‐induced HPA axis responses: Approximately 15% of chronic stress individuals show blunted GC secretion rather than elevation, and the underlying molecular mechanisms (e.g., GR polymorphism and epigenetic regulation) remain unclear, which is a key research gap. In cardiovascular disease, GCs promote angiotensin II production and increase vascular smooth muscle sensitivity to vasoconstrictors, leading to sustained hypertension—but the synergistic effect of HPA axis dysfunction and other pathways (e.g., ANS) on cerebrovascular disease progression lacks large‐sample cohort study evidence. Additionally, the role of HPA axis dysfunction in pediatric and geriatric psychosomatic diseases is understudied, with few age‐specific intervention strategies.

GC‐induced neuronal damage in the hippocampus (e.g., reduced neurogenesis and synaptic loss) is a key mechanism for cognitive and emotional regulation impairment, and preclinical studies have identified neuroprotective targets (e.g., brain‐derived neurotrophic factor and BDNF)—but translation to clinical nursing interventions (e.g., combined psychological and nutritional interventions) is still in the exploratory stage. GCs also accelerate lipolysis and alter lipid distribution, exacerbating central obesity and metabolic syndrome, but the causal relationship between GC levels and metabolic dysfunction requires further confirmation by Mendelian randomization studies.

### 3.2. ANS Imbalance Pathway

The ANS consists of the sympathetic and parasympathetic nervous systems, whose dynamic balance is the foundation of bodily physiological stability. The sympathetic nervous system follows a two‐neuron efferent pathway, where preganglionic fibers release acetylcholine (ACh) that acts on nicotinic receptors of postganglionic neurons; postganglionic fibers release norepinephrine (NE) as the primary neurotransmitter (except for sweat glands, which are innervated by postganglionic sympathetic fibers that release ACh). The parasympathetic nervous system also adopts a two‐neuron pathway, with both pre‐ and postganglionic fibers secreting ACh, which acts on nicotinic receptors of postganglionic neurons and muscarinic receptors of target organs, respectively.

**FIGURE 2:**
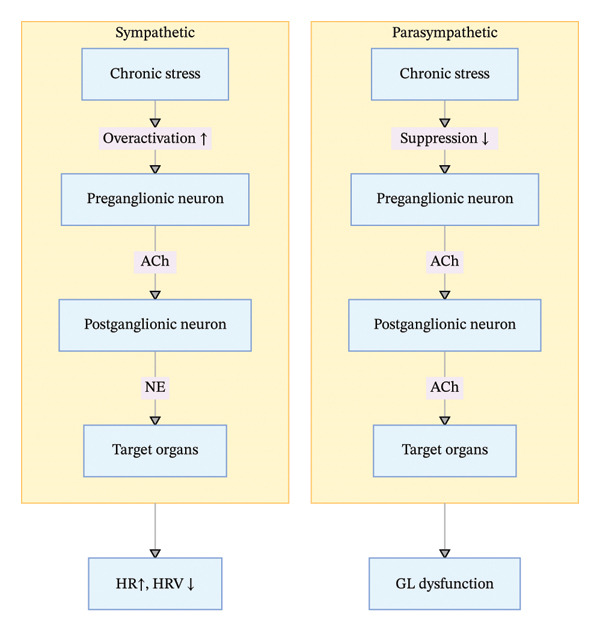
ANS imbalance pathway in chronic stress.

Under chronic stress, sympathetic nervous system activity is abnormally enhanced, while parasympathetic function is significantly suppressed—this dysregulated state is defined as autonomic nervous dysfunction [12–15] (Figure 2).

Sympathetic hyperactivation promotes massive release of NE and epinephrine (E), which bind to adrenergic receptors on target organs to trigger a series of pathological reactions. In the cardiovascular system, this leads to increased heart rate, enhanced myocardial contractility, and peripheral vasoconstriction, resulting in decreased heart rate variability (HRV), sustained hypertension, and long‐term complications such as arrhythmias and coronary insufficiency. Clinical cohort studies (e.g., the Ohasama study) have confirmed that persistent stress‐induced HRV reduction is an independent risk factor for hypertension, with a positive correlation between the degree of HRV decline and hypertension risk [14–16].

In the digestive tract, ANS imbalance disrupts gastrointestinal motility rhythms, gastric acid secretion, and intestinal microecological balance—sympathetic overactivity causes defecation difficulties, while parasympathetic dysfunction leads to excessive gastric acid secretion and mucosal barrier damage, which are key pathogenic factors for functional gastrointestinal disorders (FGIDs) such as gastroesophageal reflux disease (GERD) and irritable bowel syndrome (IBS). Notably, ANS dysfunction not only impairs local organ function but also disrupts the coordination of the systemic immune defense system by regulating immune cell activity (e.g., macrophage polarization), increasing the susceptibility to infectious diseases.

Critical evidence evaluation and research gaps: A large number of cross‐sectional studies have confirmed the correlation between ANS imbalance and chronic stress‐related psychosomatic diseases, but longitudinal interventional studies are lacking to verify the causal relationship between restoring ANS balance and improving disease prognosis. For example, aerobic exercise can enhance parasympathetic function, but the optimal exercise intensity, duration, and frequency for different populations (e.g., elderly hypertensive patients and young patients with IBS) have not been standardized. Additionally, the molecular mechanisms by which ANS imbalance regulates the gut–brain axis remain unclear, and the potential of targeted gut microbiota intervention to improve ANS function is a promising but understudied research direction. The role of ANS in chronic stress‐related reproductive system disorders (e.g., male sperm motility reduction and female menstrual cycle disorders) is also only supported by preliminary clinical evidence, with no large‐sample studies to confirm the regulatory effect of ANS‐targeted interventions.

### 3.3. Abnormal Inflammatory Response Pathway

**FIGURE 3:**
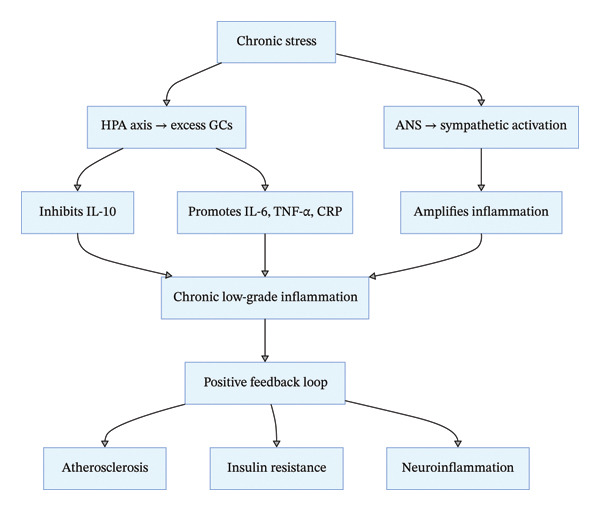
Chronic stress‐induced inflammatory response pathway.

Chronic stress induces systemic low‐grade inflammation by modulating immune cell function and inflammatory signaling pathways, which is a key bridge linking psychological stress to multisystem psychosomatic diseases [17–20] (Figure 3). Under normal physiological conditions, GCs secreted by the HPA axis exert anti‐inflammatory effects by inhibiting the activation of nuclear factor‐κB (NF‐κB) and reducing the release of proinflammatory factors. However, sustained high GC levels in chronic stress inhibit the proliferation and differentiation of macrophages and T lymphocytes, reduce the production of anti‐inflammatory mediators (e.g., IL‐10), and paradoxically promote the release of proinflammatory factors (e.g., IL‐6, TNF‐α, and CRP [18, 19]). Meanwhile, ANS imbalance exacerbates inflammatory responses: sympathetic neurotransmitter NE binds to β‐adrenergic receptors on immune cells, further amplifying the activation of inflammatory signaling pathways and forming a stress‐inflammation positive feedback loop.

Systemic low‐grade inflammation induces tissue and organ damage through multiple pathways: In the cardiovascular system, proinflammatory factors cause vascular endothelial cell injury, platelet aggregation, and lipid deposition, accelerating the progression of atherosclerosis; in the metabolic system, inflammatory mediators interfere with the insulin signaling pathway, reduce insulin receptor sensitivity, and promote the development of Type 2 diabetes; and in the nervous system, persistent inflammation impairs the integrity of the blood–brain barrier, allowing inflammatory substances to infiltrate the central nervous system, trigger neuroinflammation, and induce neuronal degeneration—this is an important pathogenic mechanism for neurodegenerative diseases (e.g., Alzheimer’s disease and Parkinson’s disease) and mood disorders (e.g., depression). Additionally, chronic stress‐induced abnormal inflammation is closely associated with the onset of autoimmune diseases (e.g., rheumatoid arthritis and psoriasis) by disrupting immune tolerance [17, 20–22].

Critical evidence evaluation and research gaps: Meta‐analyses have confirmed that chronic stress is associated with elevated peripheral blood proinflammatory factor levels, but the specificity of inflammatory factor profiles for different stress‐related psychosomatic diseases is unclear—for example, whether IL‐6 is a universal marker or a disease‐specific marker needs to be verified by large‐scale multicenter studies. The “GC resistance” phenomenon in chronic stress (i.e., high GC levels fail to exert anti‐inflammatory effects) is a key research focus, but its underlying molecular mechanisms (e.g., GR desensitization and epigenetic modification) have not been fully elucidated, and there are no clinical nursing interventions targeting GC resistance to date. Additionally, the role of local tissue inflammation (e.g., neuroinflammation and intestinal inflammation) in stress‐related diseases is more critical than systemic inflammation, but the methods for detecting local tissue inflammation in clinical practice are limited, which restricts the implementation of precise anti‐inflammatory interventions.

### 3.4. Overactive Oxidative Stress Pathway

**FIGURE 4:**
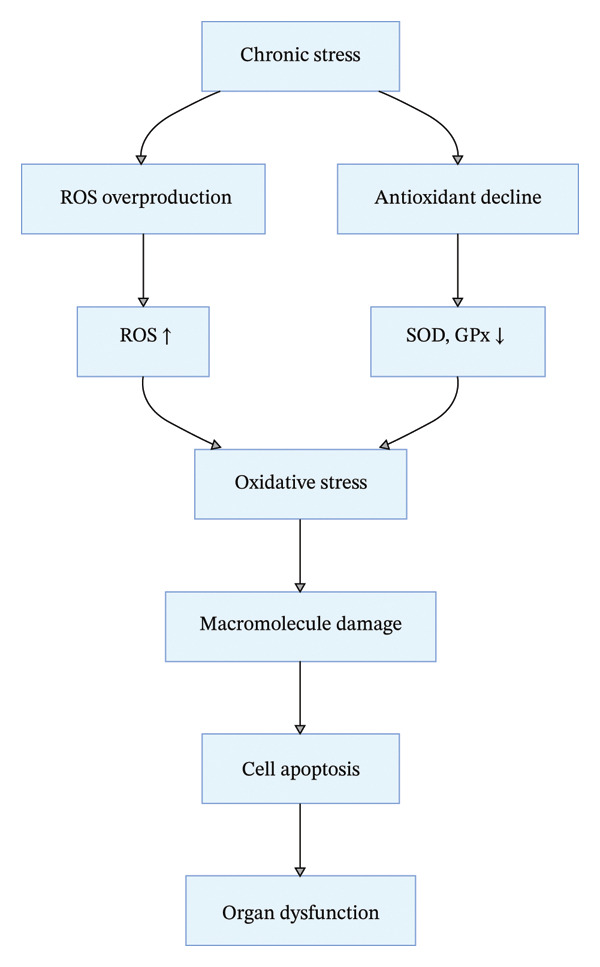
Chronic stress‐induced oxidative stress pathway.

Oxidative stress refers to the imbalance between reactive oxygen species (ROS) production and antioxidant defense capacity in the body, leading to excessive ROS accumulation and cellular damage. Chronic stress exacerbates oxidative stress through multiple interconnected pathways [23–26] (Figure 4): on the one hand, HPA axis dysfunction and ANS imbalance increase mitochondrial electron transport chain leakage, promoting massive production of ROS (e.g., superoxide anion and hydrogen peroxide); on the other hand, persistent psychological stress inhibits the activity of key antioxidant enzymes (e.g., superoxide dismutase [SOD] and glutathione peroxidase [GPx]) and reduces the content of nonenzymatic antioxidants (e.g., vitamin C and vitamin E), weakening the body’s antioxidant defense system [23, 24, 26, 27].

Excessive ROS with strong oxidizing properties causes damage to intracellular biomacromolecules (proteins, lipids, DNA), impairs normal cellular physiological functions, and induces apoptosis, leading to multisystem damage: In the cardiovascular system, ROS oxidizes low‐density lipoprotein (LDL) to form oxidized LDL (ox‐LDL), which promotes foam cell formation and accelerates atherosclerotic plaque development; in the nervous system, ROS disrupts neuronal membrane structure, induces oxidative damage to hippocampal neurons, and is a key mechanism for stress‐related depression and cognitive impairment; and in the skin, excessive oxidative stress damages collagen and elastic fibers, accelerates skin aging, and increases the risk of skin malignant tumors. Additionally, oxidative stress in the reproductive system leads to reduced male sperm motility and abnormal female ovarian function, and in the metabolic system, it exacerbates insulin resistance and metabolic syndrome progression [25, 28].

Critical evidence evaluation and research gaps: Preclinical studies have fully confirmed the role of oxidative stress in chronic stress‐related diseases, and antioxidant interventions (e.g., vitamin supplementation and exercise) have shown beneficial effects in animal models. However, clinical trials of antioxidant interventions have yielded inconsistent results—for example, high‐dose vitamin E supplementation does not reduce the risk of stress‐related cardiovascular diseases, which may be related to the lack of targeted interventions for different populations and the neglect of the synergistic effect of oxidative stress with other pathways (e.g., inflammation). The interaction between oxidative stress and epigenetic modification (e.g., DNA methylation and histone acetylation) in chronic stress is a new research direction, but the specific regulatory mechanisms and clinical translation potential are still unclear [29]. Additionally, there are no standardized clinical indicators for evaluating the degree of oxidative stress in chronic stress patients, and the combination of multiple indicators (e.g., SOD activity, malondialdehyde [MDA] levels, and GPx activity) needs to be further optimized to improve the accuracy of clinical assessment.

### 3.5. Interactions Among Biological Pathways

**FIGURE 5:**
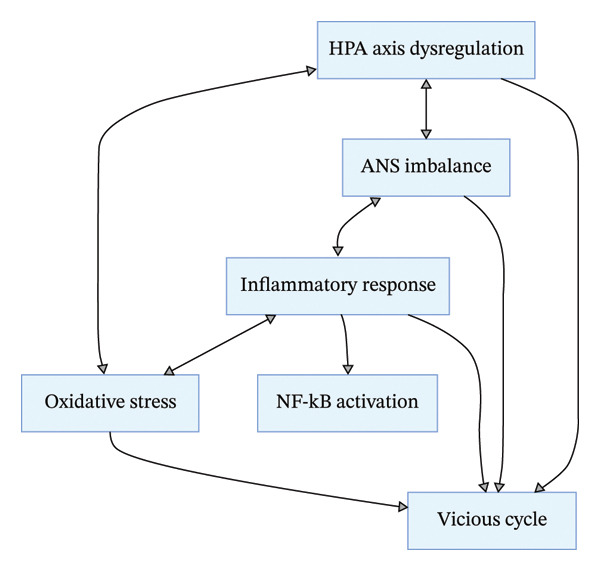
Regulatory network of interactions among chronic stress–related biological pathways.

The bodily damage caused by chronic stress is not mediated by a single biological pathway, but by the interconnected, mutually reinforcing regulatory network formed by HPA axis dysfunction, ANS imbalance, abnormal inflammatory responses, and excessive oxidative stress [7, 30] (Figure 5). Key interaction mechanisms are as follows:

1. HPA axis dysfunction promotes oxidative stress: Sustained high GC levels inhibit the activity of antioxidant enzymes (e.g., SOD and GPx), reduce the body’s antioxidant capacity, and exacerbate ROS accumulation;
2. Oxidative stress amplifies inflammatory responses: Excessive ROS activates the NF‐κB inflammatory signaling pathway, promotes the release of proinflammatory factors, and forms an oxidative stress‐inflammation positive feedback loop;
3. ANS imbalance exacerbates HPA axis dysfunction: Sympathetic neurotransmitter NE binds to receptors on hypothalamic and pituitary cells, enhancing HPA axis activity and maintaining sustained high GC levels;
4. Inflammation and oxidative stress jointly damage the HPA axis and ANS: Persistent inflammation and excessive ROS cause damage to hypothalamic, pituitary, and autonomic nerve neurons, further impairing the regulatory function of these systems and forming a multipathway vicious cycle.

Clinical evidence confirms that individuals under prolonged chronic stress have a significantly higher incidence of multisystem comorbidities (e.g., hypertension + depression + metabolic syndrome) than the general population, which is closely related to the cumulative effects of the above pathway interactions. For example, hypertension induced by HPA axis and ANS dysfunction is further exacerbated by inflammation‐induced vascular endothelial damage and oxidative stress‐induced atherosclerotic progression; depression caused by hippocampal neuronal damage is aggravated by neuroinflammation and oxidative stress, and in turn, depression further enhances stress perception, forming a psychosomatic vicious cycle [7, 30].

Critical evidence evaluation and research gaps: Current research has clarified the basic interaction mechanisms among individual pathways, but the overall regulatory network of multiple pathways lacks quantitative research and predictive models—for example, there is no clear understanding of the weight of each pathway in the onset of different psychosomatic diseases, which restricts the development of targeted combined intervention strategies. Additionally, the molecular “crosstalk nodes” among pathways (e.g., NF‐κB, which is regulated by both oxidative stress and ANS) are potential intervention targets [30], but preclinical studies on single‐target interventions have shown limited efficacy, and the research on multitarget combined interventions is still in the initial stage. The dynamic changes of pathway interactions at different stages of chronic stress (e.g., early stress, persistent stress, and stress recovery) are also unclear, and there is a lack of stage‐specific intervention strategies.

### 3.6. Definition and Model of Chronic Stress as a “Risk Hub”

**FIGURE 6:**
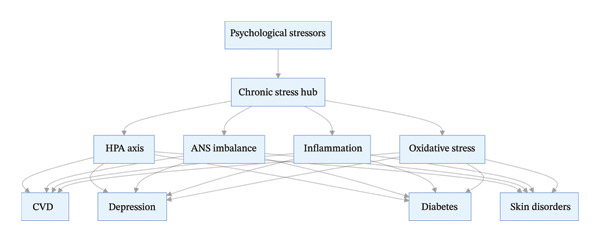
Chronic stress as a risk hub for psychosomatic disease model.

Definition: Chronic stress acts as a central “risk hub” in the pathogenesis of psychosomatic diseases, which receives and integrates multiple psychological stress signals (e.g., work pressure, life events, and emotional distress), and transduces these signals into physiological and pathological changes through the above four core biological pathways and their interaction network. This hub not only mediates the onset of a single psychosomatic disease but also promotes the comorbidity of multiple diseases by regulating common biological pathways, and amplifies the disease risk through positive feedback loops between pathways and between the mind and body [31, 32] (Figure 6).

The “risk hub” model has three core characteristics [33–38]:

1. Signal integration: The hub integrates diverse psychological stress signals (quantitative and qualitative) and converts them into unified biological signals (e.g., GC elevation, NE release, and proinflammatory factor secretion);
2. Pathway divergence: The integrated biological signals are transmitted to different target organs/systems through multiple core biological pathways, leading to the onset of diverse psychosomatic diseases (e.g., cardiovascular system, nervous system, and metabolic system);
3. Comorbidity promotion: The interaction network among pathways enables the risk hub to regulate multiple systems simultaneously, leading to multisystem comorbidities, and the vicious cycle between the mind and body further amplifies the disease risk.

The clinical significance of the “risk hub” model is that it provides a new theoretical basis for precise clinical nursing: Clinical nurses can identify the activation state of the risk hub and the dominant pathological pathways through comprehensive assessment (physiological parameters + psychosocial factors), and develop personalized intervention plans targeting the hub and dominant pathways, rather than adopting generalized symptomatic interventions. For example, for patients with chronic stress combined with hypertension and depression, the intervention should focus on inhibiting the activation of the risk hub (reducing stress perception) and targeting the dominant pathways (regulating HPA axis and ANS, inhibiting neuroinflammation).

## 4. Integration of Cutting‐Edge Research Advances

### 4.1. Peripheral Stress Response System: Neuro‐Immuno‐Endocrinology of the Skin

Traditional research on stress responses focuses on the central neuroendocrine system (HPA axis, ANS), while recent cutting‐edge research [39] has confirmed that the skin, as the largest peripheral organ of the body, has a complete and functional neuro‐immuno‐endocrine stress response system, which is an important extension of the central stress response system and closely interacts with the central system to regulate the body’s stress response. The skin neuro‐immuno‐endocrine system can independently produce classic stress‐related mediators (e.g., CRH, ACTH, GCs, melatonin, and neuropeptides) and has corresponding receptors, forming a cutaneous HPA axis homolog that can respond to local environmental stressors (e.g., solar radiation, physical/chemical damage, and pathogens) and systemic psychological stress [39–44].

**FIGURE 7:**
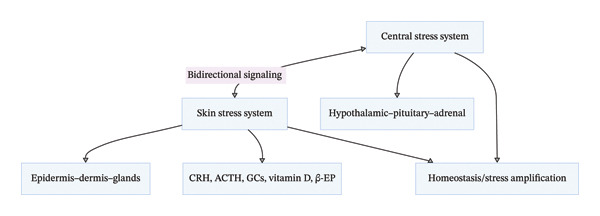
Interaction between central and skin neuro‐immuno‐endocrine stress response systems.

Chronic psychological stress can activate the skin neuro‐immuno‐endocrine system, leading to skin dysfunction and even skin diseases (e.g., psoriasis, atopic dermatitis, and skin aging); conversely, skin environmental stressors (e.g., moderate ultraviolet [UV] radiation) can regulate the central stress response system through the skin–brain axis, exerting a positive regulatory effect on chronic stress (Section 4.3). The interaction between the skin and central stress response systems is bidirectional: Central stress signals are transmitted to the skin through the ANS and systemic circulation, while skin stress signals are transmitted to the central nervous system through peripheral nerves, circulating mediators, and immune cell priming, forming a whole‐body stress response network [39–41, 44] (Figure 7).

Clinical nursing implications: For chronic stress patients with concomitant skin diseases (e.g., stress‐induced psoriasis), nursing interventions should not only target the central stress response system but also regulate the skin neuro‐immuno‐endocrine system—for example, maintaining skin barrier integrity, avoiding skin environmental stressors (e.g., excessive UV radiation and chemical irritation), and using topical agents that regulate skin inflammatory and oxidative stress levels [45–47]. Additionally, skin can be used as a peripheral intervention target for chronic stress: Moderate skin stimulation (e.g., massage and phototherapy) can activate the skin’s stress response regulatory system and transmit positive regulatory signals to the central nervous system to alleviate stress perception.

### 4.2. Stress–Cancer Crosstalk: Neuroendocrine Regulation of Cancer Homeostasis

Recent research [10] has confirmed that there is a mutual regulatory relationship between chronic stress and cancer—chronic stress not only increases the risk of cancer onset and progression but also impairs the efficacy of cancer treatment and reduces patient survival; conversely, cancer‐related stress (e.g., disease‐related anxiety and treatment side effects) further activates the body’s stress response system, forming a stress–cancer vicious cycle [33]. The underlying mechanisms are closely related to the four core biological pathways of chronic stress: HPA axis dysfunction‐induced GC elevation inhibits antitumor immune function (e.g., reducing cytotoxic T‐cell activity), ANS imbalance promotes tumor angiogenesis and metastasis (e.g., NE‐induced vascular endothelial growth factor [VEGF] secretion), abnormal inflammatory responses create a protumor inflammatory microenvironment, and excessive oxidative stress induces DNA damage and promotes tumor cell proliferation and mutation.

Notably, cancer cells can hijack the body’s neuroendocrine stress response system to maintain their own homeostasis: Cancer cells can produce and secrete stress‐related mediators (e.g., CRH and GCs) and express corresponding receptors, forming an autocrine/paracrine regulatory loop that promotes cancer cell proliferation, invasion, and metastasis. Additionally, cancer‐induced chronic stress impairs the body’s antitumor immune response and promotes the development of cancer‐related fatigue, depression, and other psychosomatic symptoms, which significantly reduce the quality of life of cancer patients.

Clinical nursing implications: Chronic stress management should be incorporated into the comprehensive nursing of cancer patients, and targeted stress intervention strategies should be developed according to the cancer type, treatment stage, and stress state of patients. For example, for patients with breast cancer undergoing chemotherapy, cognitive behavioral therapy (CBT) and relaxation training can be used to reduce stress perception, regulate HPA axis and ANS function, and improve antitumor immune function and treatment tolerance; for cancer patients with severe depression and anxiety, combined psychological intervention and melatonin supplementation (Section 5.1.2) can be used to alleviate stress‐related symptoms and improve the quality of life. Additionally, clinical nurses should conduct regular stress assessment for cancer patients, identify high‐risk groups of stress‐induced cancer progression, and implement early intervention.

### 4.3. Photo‐Neuro‐Immuno‐Endocrinology: Positive Effect of Moderate Solar Radiation on Chronic Stress

Most research focuses on the harmful effects of environmental stressors on the body, while recent photo‐neuro‐immuno‐endocrinology research [48, 49] has confirmed that moderate solar UV radiation (a key environmental stressor of the skin) exerts a positive regulatory effect on chronic stress through the skin–brain axis, which is an important nonpharmacological intervention for chronic stress [45–47]. The underlying mechanisms are as follows:

1. Moderate UV radiation promotes the skin to produce vitamin D, which not only regulates calcium and phosphorus metabolism but also inhibits the activation of the HPA axis, reduces GC secretion, and exerts anti‐inflammatory and antioxidant effects;
2. UV radiation stimulates the skin to produce β‐endorphin, which is transmitted to the central nervous system through the systemic circulation, binds to opioid receptors in the brain, and produces analgesic and anxiolytic effects, reducing stress perception;
3. Moderate UV radiation regulates the skin neuro‐immuno‐endocrine system, inhibits the release of proinflammatory factors, and reduces systemic low‐grade inflammation, thereby alleviating the inflammatory damage caused by chronic stress;
4. UV radiation regulates the circadian rhythm by stimulating the skin’s photosensitive cells, improving sleep quality (Section 5.1.2), and further restoring the normal regulatory function of the HPA axis.

Notably, the effect of UV radiation on chronic stress is dose‐dependent: moderate UV radiation exerts a positive regulatory effect, while excessive UV radiation causes skin damage (e.g., sunburn and skin cancer), activates the body’s stress response system, and exacerbates chronic stress. Additionally, the positive effect of UV radiation varies among populations, and factors such as skin type, age, and geographical location should be considered in clinical application [48, 49].

Clinical nursing implications: Clinical nurses can guide chronic stress patients to receive moderate solar radiation as a nonpharmacological intervention—for example, advising patients to take 15–20 min of outdoor sunlight exposure daily (9:00–10:00 or 16:00–17:00, avoiding strong noon sunlight) according to their skin type, which can improve vitamin D levels, regulate circadian rhythm, and alleviate stress perception. For patients who are unable to receive outdoor sunlight exposure (e.g., bedridden patients and office workers), artificial low‐dose UV phototherapy can be recommended under the guidance of physicians. Additionally, nurses should remind patients to take sun protection measures (e.g., wearing sun hats and applying low‐SPF sunscreen) during sunlight exposure to avoid excessive UV radiation‐induced skin damage.

## 5. Clinical Nursing Assessment: Identification of Stress‐Related Health Risks Based on Biological Pathways

Clinical nursing assessment based on biological pathways is the basis for identifying chronic stress high‐risk populations and developing precise intervention plans. The assessment system is improved in this revision by adding real‐world nursing assessment examples, optimizing risk stratification criteria, and clarifying the correlation between assessment indicators and biological pathways. The assessment includes three parts: physiological parameter evaluation, psychosocial assessment, and risk stratification assessment (Table 1), with the core principle of combining biological pathway indicators and clinical symptoms to achieve comprehensive and precise assessment [50–54].

**TABLE 1:** Chronic stress clinical nursing assessment system based on biological pathways.

Assessment domain	Key indicators	Detection methods	Clinical significance
HPA axis function	Serum cortisol (morning/night), circadian rhythm	Blood test, ELISA	Rhythm disturbance = HPA overactivation
ANS function	Heart rate variability (HRV), resting heart rate	Portable ECG, smart wristband	Low HRV = sympathetic dominance
Inflammatory status	IL‐6, TNF‐α, CRP	Blood test, POCT	Elevation = low‐grade chronic inflammation
Oxidative stress	SOD, GPx, MDA	Colorimetric assay	Antioxidant decline = oxidative damage
Psychosocial status	PSS‐10, GAD‐7, PHQ‐9	Standardized scales	Stress perception, anxiety, depression
Lifestyle and support	Sleep quality, diet, exercise, social support	Questionnaire, interview	Risk or protective factors for stress injury

### 5.1. Physiological Parameter Evaluation

Physiological parameter evaluation is used to detect the activation state of core biological pathways and the degree of target organ damage, with specific, quantifiable biological markers as the main assessment indicators, combined with traditional physiological parameters. The assessment indicators are closely linked to the four core biological pathways, and the detection methods and clinical significance are clarified as follows:

1. HPA axis function indicators: Serum cortisol concentration (morning and evening samples) and circadian rhythm analysis—nocturnal cortisol peak elevation is an important biological marker of chronic stress; salivary cortisol detection is recommended for clinical nursing due to its noninvasiveness and convenience.
2. ANS function indicators: HRV analysis—reduced HRV is a marker of sympathetic hyperactivation and an early warning indicator of stress‐related cardiovascular diseases; HRV can be detected by portable devices (e.g., smart wristbands) for long‐term dynamic monitoring.
3. Inflammatory state indicators: Peripheral blood proinflammatory factors (IL‐6, TNF‐α) and inflammatory markers (CRP)—elevated levels indicate chronic low‐grade inflammation; point‐of‐care testing (POCT) can be used for rapid detection in clinical nursing [51, 54].
4. Oxidative stress indicators: Antioxidant enzyme activity (SOD, GPx) and oxidative damage products (MDA)—reduced SOD/GPx activity and elevated MDA levels indicate excessive oxidative stress.
5. Traditional physiological parameters: Blood pressure, heart rate, body mass index (BMI), blood glucose, blood lipids—used to assess the degree of target organ damage (e.g., cardiovascular and metabolic system) caused by chronic stress.

Real‐world nursing example: A 35‐year‐old female office worker with a 2‐year history of work pressure complains of persistent anxiety, sleep disturbance, and occasional headache. Nursing assessment: serum cortisol (nocturnal peak 2.1 times the normal value), HRV (SDNN < 50 ms), CRP (8.2 mg/L, normal < 5 mg/L), and SOD activity (110 U/mL, normal 120–200 U/mL). The results indicate HPA axis dysfunction, ANS imbalance, mild chronic inflammation, and reduced antioxidant capacity—suggesting moderate stress‐related health risks, with potential early damage to the cardiovascular and nervous systems.

### 5.2. Psychosocial Assessment

Psychosocial assessment is used to identify stress sources, stress perception intensity, emotional state, and social support status, and adopts standardized scales + structured interviews to achieve a quantitative and qualitative combination assessment. The revised section optimizes the scale selection, clarifies the assessment content, and links the assessment results to the activation of the chronic stress “risk hub”:

1. Stress assessment scales: Perceived Stress Scale (PSS‐10), Life Event Scale (LES)—evaluate stressor characteristics (e.g., work, life, and interpersonal relationships), perceived stress intensity, and cumulative stress load; PSS‐10 score > 20 indicates high perceived stress intensity.
2. Emotional state scales: Self‐Rating Depression Scale (SDS), Self‐Rating Anxiety Scale (SAS), Generalized Anxiety Disorder‐7 (GAD‐7)—quantitatively evaluate stress‐related emotional symptoms (depression, anxiety); SDS standard score > 53 and SAS standard score > 50 indicate mild to moderate depression/anxiety [55, 56].
3. Structured interviews: Collect information on social support network (family relationships, social interactions, social resource utilization), stress coping strategies (active/passive coping), and lifestyle habits (sleep pattern, diet, exercise)—evaluate the ability to cope with stress and potential modifiable risk factors [50, 52, 53, 57].

Real‐world nursing example: For the above 35‐year‐old female office worker, psychosocial assessment shows PSS‐10 score 26, SAS standard score 62, LES score 180 (cumulative high work pressure), social support score 32 (low social support), and passive coping strategies (overthinking, avoidance). The results indicate high stress perception intensity, moderate anxiety, low social support, and ineffective coping strategies—these factors are important drivers of the activation of the chronic stress “risk hub.”

### 5.3. Risk Stratification Assessment

Risk stratification assessment is based on the comprehensive results of physiological parameter evaluation and psychosocial assessment, and divides chronic stress‐related health risks into low‐, moderate‐, and high‐risk groups with clear stratification criteria [51, 58] (Table 2). The revised section refines the stratification indicators, links the risk level to the activation state of the “risk hub” and the dominant biological pathways, and provides targeted nursing intervention directions for each risk group to achieve stratified and precise nursing.

1. Low‐risk group: Mild stress perception (PSS‐10 ≤ 20), all physiological indicators (cortisol, HRV, inflammatory factors, oxidative stress) within normal ranges, no significant emotional symptoms, and effective stress coping strategies + good social support. Nursing intervention direction: health education, lifestyle guidance, and regular follow‐up to prevent stress escalation.
2. Moderate‐risk group: Moderate stress perception (20 < PSS‐10 ≤ 30), partial physiological indicators abnormal (e.g., nocturnal cortisol elevation, mild HRV reduction, and slight CRP elevation), mild emotional symptoms (SDS/SAS standard score 53–69) that do not meet clinical diagnostic criteria, and relatively insufficient social support/ineffective coping strategies. Nursing intervention direction: targeted psychological intervention (e.g., relaxation training), lifestyle modification (e.g., sleep intervention and exercise guidance), and dynamic monitoring of physiological indicators.
3. High‐risk group: Severe stress perception (PSS‐10 > 30), multiple physiological indicators significantly abnormal (e.g., persistent cortisol elevation, severe HRV reduction, and high inflammatory factor levels), moderate to severe emotional symptoms (SDS/SAS standard score ≥ 70), or confirmed diagnosis of psychosomatic diseases (e.g., hypertension, depression, and Type 2 diabetes), and poor social support/maladaptive coping strategies. Nursing intervention direction: comprehensive combined intervention (psychological intervention + pharmacological intervention under physician guidance + pathway‐specific nursing), close monitoring of disease progression, and multidisciplinary collaboration (nursing, psychology, clinical medicine).

**TABLE 2:** Chronic stress‐related health risk stratification criteria.

Risk level	Perceived stress (PSS‐10)	Physiological indicators	Psychological symptoms (SAS/SDS)	Social support and coping	Nursing intervention
Low	≤ 20	All within normal range	< 53 No obvious symptoms	Adequate, effective	Health education, lifestyle guidance
Moderate	21–30	1–2 abnormalities (e.g., mild HRV ↓, CRP ↑)	53–69 Mild symptoms	Insufficient	Mindfulness, breathing training, anti‐inflammatory diet
High	> 30	Multiple marked abnormalities + comorbidities	≥ 70 Moderate–severe symptoms	Poor, maladaptive	CBT, multidisciplinary care, intensive monitoring

## 6. Clinical Nursing Intervention Strategies Based on Biological Pathways

The core revision of this section is to strengthen the mechanistic link between nursing interventions and biological pathway modulation, supplement pathway‐specific intervention details, add real‐world nursing intervention examples, and integrate cutting‐edge research advances (e.g., melatonin supplementation, skin‐targeted intervention, and moderate UV radiation). The intervention strategies are developed according to the four core biological pathways and their interaction network, with the principles of targeting the dominant pathway, combining multiple interventions, and personalized implementation. All interventions are clearly linked to the corresponding biological pathway mechanisms, avoiding generalized and nonspecific intervention measures, and realizing pathway‐targeted precise clinical nursing.

### 6.1. Nursing Interventions for Regulating HPA Axis Function

The core goal of intervention is to restore the normal negative feedback regulatory function of the HPA axis, reduce sustained high GC levels, and alleviate GC‐induced multisystem damage. Interventions are divided into psychological interventions, sleep care (supplemented with melatonin application), and lifestyle guidance, with clear mechanistic links to HPA axis regulation and specific implementation methods.

#### 6.1.1. Psychological Intervention

CBT is the first‐line psychological intervention for regulating HPA axis function, with the mechanism of guiding patients to identify and modify negative thought patterns, establish rational belief systems, and reduce stress perception—thereby inhibiting HPA axis hyperactivity and restoring normal cortisol circadian rhythm. Clinical evidence confirms that CBT can significantly reduce serum cortisol levels in chronic stress populations, improve hippocampal neuronal activity, and alleviate anxiety and depression symptoms [59].

Implementation methods: Conduct individual CBT by clinical nurses (with psychological intervention training) 1–2 times a week, 45–60 min each time, for a total of 8–12 sessions; group CBT (5–8 patients) can also be conducted to improve intervention efficiency. Relaxation techniques (mindfulness meditation, progressive muscle relaxation) are used as adjuvant interventions: guide patients to practice 15–20 min twice a day (morning and evening), with the mechanism of inhibiting hypothalamic CRH secretion and reducing pituitary ACTH release, thereby suppressing HPA axis hyperactivity.

#### 6.1.2. Sleep Care

Sleep disorders and HPA axis dysfunction form a bidirectional vicious cycle: Sleep disorders exacerbate HPA axis dysfunction, and HPA axis hyperactivity (nocturnal cortisol elevation) further impairs sleep quality. The core goal of sleep care is to restore normal circadian rhythm and improve sleep quality, thereby restoring the normal negative feedback regulation of the HPA axis. The revised section adds melatonin application (an important regulator of circadian rhythm and HPA axis function) and clarifies its clinical nursing application methods.

Basic sleep intervention: Conduct a comprehensive sleep assessment for patients and develop individualized sleep intervention plans; guide patients to establish a regular sleep schedule (fixed bedtime and wake‐up time) and create a comfortable sleep environment (appropriate temperature, soft lighting, quiet); advise patients to reduce electronic device usage 1 h before bedtime (avoid blue light interference) and avoid strenuous exercise and stimulant intake (caffeine, alcohol, spicy food) [60–62].

Melatonin supplementation intervention: For patients with moderate to severe sleep disturbance (e.g., insomnia and delayed sleep phase), melatonin supplementation is recommended under physician guidance—melatonin inhibits HPA axis hyperactivity by binding to melatonin receptors in the hypothalamus, reduces nocturnal cortisol secretion, and regulates circadian rhythm. Nursing guidance: Advise patients to take low‐dose melatonin (0.5–3 mg) 30–60 min before bedtime, avoid high‐dose long‐term use; monitor sleep quality and serum cortisol levels during supplementation, and adjust the dose according to the physician’s advice. For patients who refuse drug supplementation, guide them to increase dietary melatonin intake (e.g., cherries, walnuts, and oats) [63–65].

Real‐world nursing example: For the 35‐year‐old female office worker with moderate stress risk, implement 8 sessions of individual CBT + daily mindfulness meditation (20 min) + sleep intervention (regular sleep schedule + low‐dose melatonin supplementation 1 mg before bedtime). After 4 weeks of intervention, nursing reassessment shows nocturnal cortisol peak reduced to 1.2 times the normal value, sleep quality significantly improved, and SAS standard score reduced to 51.

#### 6.1.3. Lifestyle Guidance

Guide patients to avoid lifestyle factors that activate the HPA axis (e.g., irregular work and rest, excessive work load, and long‐term mental tension); advise patients to arrange work and rest time reasonably, take regular breaks (5–10 min every 1–2 h of work), and avoid overwork; encourage patients to participate in pleasant activities (e.g., listening to music, reading, and gardening) to reduce mental tension and inhibit HPA axis hyperactivity.

### 6.2. Nursing Interventions for Improving ANS Imbalance

The core goal of intervention is to restore the dynamic balance between the sympathetic and parasympathetic nervous systems, inhibit sympathetic hyperactivation, and enhance parasympathetic function. Interventions are divided into exercise guidance and respiratory training, with the mechanism of action clearly linked to ANS regulation, and personalized implementation based on patient characteristics (age, health status, disease type).

#### 6.2.1. Exercise Guidance

Moderate exercise is the first‐line intervention for improving ANS imbalance, with the mechanism of enhancing parasympathetic nerve function, inhibiting sympathetic hyperactivation, and increasing HRV—thereby restoring ANS dynamic balance. The revised section standardizes exercise intensity, duration, and type, and adds exercise recommendations for different populations.

General exercise recommendations: Low‐to‐moderate‐intensity aerobic exercise (brisk walking, jogging, swimming, cycling) is recommended, with a weekly cumulative duration of no less than 150 min, 30 min each time, 5 times a week; mind‐body integrated exercises (Tai Chi, yoga, qigong) are recommended as adjuvant exercises, 2–3 times a week, 40–60 min each time—clinical evidence confirms that Tai Chi and yoga can significantly increase HRV and improve ANS regulatory function [66].

Population‐specific exercise recommendations: For elderly patients with stress‐related hypertension, low‐intensity aerobic exercise (slow walking, Tai Chi) is recommended to avoid excessive exercise‐induced cardiovascular burden; for young patients with stress‐induced IBS, moderate‐intensity aerobic exercise (jogging, cycling) is recommended to improve gastrointestinal motility and ANS function; for bedridden patients, passive limb exercise and bed yoga are implemented by nurses to maintain ANS function.

Nursing implementation: Conduct a comprehensive physical assessment for patients and develop individualized exercise plans; guide patients to record exercise logs, and monitor exercise intensity (heart rate maintained at 60%–70% of maximum heart rate) and exercise effects; and conduct regular follow‐up and adjust the exercise plan according to the patient’s condition.

#### 6.2.2. Respiratory Training

Respiratory training regulates ANS function by adjusting respiratory rate and depth, with the core mechanism of activating the parasympathetic nervous system (vagus nerve) and inhibiting sympathetic hyperactivation—thereby increasing HRV and reducing stress‐related physiological responses (e.g., increased heart rate and elevated blood pressure). The revised section standardizes respiratory training methods and implementation steps, and clarifies the clinical application scope [67, 68].

Recommended training methods: Diaphragmatic breathing and slow low‐frequency breathing (6–8 breaths per minute) are the main methods, with the following implementation steps: 1. The patient takes a comfortable sitting/lying position, relaxes the whole body; 2. inhales deeply through the nose for 4–6 s, allowing the abdomen to rise gradually; 3. holds the breath for 1–2 s; 4. exhales slowly through the mouth for 6–8 s, allowing the abdomen to retract to the original position; 5. repeats 10–15 times per training session, 2–3 times a day (morning, noon, evening).

Nursing implementation: Nurses demonstrate respiratory training methods to patients face‐to‐face, correct incorrect movements in real time; guide patients to practice with the help of audio/video materials for self‐training at home; for patients with stress‐related cardiovascular diseases (e.g., hypertension and arrhythmias), combine respiratory training with exercise guidance to improve ANS regulation effect.

Real‐world nursing example: A 45‐year‐old male patient with chronic work pressure, diagnosed with mild hypertension (145/95 mmHg), HRV (SDNN 45 ms). Nursing intervention: moderate‐intensity brisk walking (30 min a day, 5 times a week) + diaphragmatic breathing training (15 min a day, 3 times a week). After 8 weeks of intervention, reassessment shows blood pressure reduced to 135/85 mmHg, HRV (SDNN 65 ms), and ANS function significantly improved.

### 6.3. Nursing Interventions for Chronic Inflammation Suppression

The core goal of intervention is to inhibit chronic stress‐induced systemic low‐grade inflammation, break the stress‐inflammation positive feedback loop, and reduce inflammatory damage to target organs. Interventions are divided into dietary care and social support interventions, with the mechanism of action linked to inflammatory regulation, and specific implementation methods and real‐world examples added.

#### 6.3.1. Dietary Care

Balanced anti‐inflammatory diet regulates immune cell function and inhibits inflammatory signaling pathways (e.g., NF‐κB), thereby reducing the release of proinflammatory factors and alleviating chronic low‐grade inflammation. The revised section refines the anti‐inflammatory diet plan, clarifies the recommended food types and intake, and links the dietary components to anti‐inflammatory mechanisms [69].

Anti‐inflammatory diet core recommendations:

1. Increase intake of ω‐3 polyunsaturated fatty acids: Deep‐sea fish (salmon, mackerel, sardines) 2–3 times a week, flaxseed oil, walnuts—ω‐3 fatty acids block the synthesis of proinflammatory mediators (e.g., prostaglandin E2) and exert anti‐inflammatory effects [70].
2. Increase intake of antioxidant‐rich foods: Fresh fruits and vegetables (blueberries, strawberries, spinach, broccoli), whole grains (oats, quinoa, brown rice)—antioxidant components (vitamin C, vitamin E, polyphenols) eliminate ROS, reduce oxidative stress, and further mitigate inflammation [71].
3. Reduce intake of proinflammatory foods: High‐sugar, high‐fat, processed foods (pastries, fried foods, processed meat), refined carbohydrates (white rice, white bread)—these foods promote the release of proinflammatory factors and exacerbate chronic inflammation.
4. Moderate intake of probiotics: Yogurt, kefir, kimchi—regulate intestinal microecological balance, improve intestinal barrier function, and reduce intestinal inflammation‐induced systemic inflammation (gut–brain axis regulation).

Nursing implementation: Conduct a dietary assessment for patients and understand dietary habits and food preferences; develop individualized anti‐inflammatory diet plans based on patient characteristics (e.g., vegetarian and food allergy); guide patients to record diet logs, monitor dietary compliance; and regular follow‐up and adjust the diet plan according to the patient’s inflammatory indicator levels (e.g., CRP and IL‐6) [72, 73].

#### 6.3.2. Social Support Intervention

Sufficient social support alleviates chronic stress perception by providing emotional comfort and practical assistance, thereby reducing stress‐induced inflammatory responses—breaking the stress‐inflammation positive feedback loop. The revised section optimizes the social support intervention plan, clarifies the intervention methods, and adds the establishment of peer support groups [74, 75].

Social support intervention methods:

1. Evaluate and optimize social support network: Conduct a comprehensive assessment of the patient’s social support status (family, friends, and colleagues) and identify insufficient social support links (e.g., poor family relationships and lack of friend communication); guide patients to strengthen communication with family and friends, express psychological feelings and needs, and obtain emotional comfort and practical assistance.
2. Establish peer support groups: Organize chronic stress patients to form peer support groups (5–10 people), conduct regular exchange activities (1–2 times a week)—patients share stress coping experiences and feelings, provide mutual emotional support and practical guidance, and expand social support channels.
3. Refer to professional social support services: For patients with severe family conflicts or interpersonal relationship problems, refer to professional family counseling or interpersonal relationship improvement services to improve social support capacity.

Real‐world nursing example: A 50‐year‐old female patient with chronic care stress for a family member, diagnosed with moderate depression, CRP (10.5 mg/L). Nursing intervention: anti‐inflammatory diet plan (2 times a week deep‐sea fish + daily fresh fruits and vegetables + moderate probiotic intake) + social support intervention (guide to strengthen communication with siblings, join a caregiver peer support group). After 6 weeks of intervention, reassessment shows CRP reduced to 6.1 mg/L, depression symptoms significantly alleviated, and stress perception intensity reduced.

### 6.4. Nursing Interventions to Alleviate Oxidative Stress

The core goal of intervention is to restore the balance between ROS production and antioxidant defense capacity, reduce excessive ROS accumulation, and alleviate oxidative damage to biomacromolecules and target organs. Interventions are divided into antioxidant supplementation guidance and adverse lifestyle intervention, with the mechanism of action linked to oxidative stress regulation, and the intervention details are standardized and personalized.

#### 6.4.1. Supplementary Guidelines for Antioxidant Supplementation

The core principle is dietary supplementation as the main method, and drug supplementation as the adjuvant method—guide patients to obtain antioxidant components from natural foods to improve the body’s antioxidant capacity, and avoid high‐dose long‐term drug supplementation. The revised section clarifies the main antioxidant components, their food sources, and the clinical application of drug supplementation, and links the antioxidant components to oxidative stress regulation mechanisms [76, 77].

Key antioxidant components and food sources:

1. Vitamin C: Fresh fruits and vegetables (citrus, kiwi, bell pepper, spinach)—scavenges free radicals, regenerates vitamin E, and enhances antioxidant capacity.
2. Vitamin E: Vegetable oils (olive oil, sunflower oil), nuts (almonds, walnuts), avocados—inhibits lipid peroxidation, protects cell membranes from oxidative damage.
3. Selenium: Seafood (shrimp, crab, kelp), whole grains, eggs—component of GPx, enhances GPx activity.
4. Polyphenols: Green tea, dark chocolate, blueberries, grapes—scavenges ROS, inhibits oxidative stress‐induced inflammatory signaling pathway activation.

Drug supplementation guidance: For patients with severe oxidative stress (e.g., significantly reduced SOD/GPx activity and elevated MDA levels), antioxidant drug supplementation is recommended under physician guidance (e.g., vitamin C tablets, vitamin E soft capsules, and selenium yeast tablets); nurses guide patients to take the drugs according to the dose, monitor antioxidant indicator levels during supplementation, and adjust the dose according to the physician’s advice; remind patients of the potential hazards of high‐dose supplementation (e.g., vitamin C overdose causes gastrointestinal discomfort and vitamin E overdose causes bleeding risk).

#### 6.4.2. Adverse Lifestyle Intervention

Unhealthy lifestyle habits (smoking, excessive alcohol consumption, sedentary behavior) significantly increase ROS production and exacerbate oxidative stress—intervening in these habits is an important measure to alleviate oxidative stress. The revised section standardizes the intervention methods for smoking and alcohol consumption, adds sedentary behavior intervention, and clarifies the nursing implementation steps [78–81].

1. Smoking intervention: Conduct systematic health education to inform patients of the harmful effects of smoking on oxidative stress (tobacco smoke contains a large number of free radicals, which reduce antioxidant enzyme activity); for patients with smoking cessation intentions, guide them to use nicotine replacement therapy (e.g., nicotine gum and transdermal patches) under physician guidance; conduct regular follow‐up (1, 3, 6 months) to evaluate smoking cessation efficacy, and provide psychological support for patients with smoking cessation difficulties.
2. Alcohol consumption intervention: Remind patients of the harmful effects of excessive alcohol consumption on oxidative stress (alcohol metabolism produces acetaldehyde and ROS, which cause oxidative damage); formulate a strict alcohol intake limit: men ≤ 25 g of pure alcohol per day, women ≤ 15 g of pure alcohol per day; for patients with excessive alcohol consumption, guide them to reduce alcohol intake gradually, and refer to professional alcohol abstinence services if necessary.
3. Sedentary behavior intervention: Guide patients to avoid long‐term sedentary behavior (≤ 8 h of sitting per day), take regular breaks (stand up and move for 5 min every 1 h of sitting); encourage patients to participate in moderate physical activity (Section 6.2.1) to reduce ROS production and enhance antioxidant capacity.

Real‐world nursing example: A 55‐year‐old male patient with chronic stress, long‐term smoking (20 cigarettes a day) and excessive alcohol consumption (500 mL of beer a day), SOD activity (95 U/mL), MDA (10.2 nmol/mL). Nursing intervention: antioxidant diet plan (daily citrus + almonds + spinach) + smoking cessation intervention (nicotine gum + health education) + alcohol consumption limitation (≤ 200 mL of beer a day) + moderate brisk walking (30 min a day). After 3 months of intervention, reassessment shows the patient quit smoking, alcohol consumption reduced to the limit, SOD activity (135 U/mL), MDA (7.1 nmol/mL), and oxidative stress significantly were alleviated.

### 6.5. Combined Interventions for Pathway Interaction Network

Given that chronic stress‐induced bodily damage is mediated by the interaction network of multiple biological pathways, single‐pathway intervention has limited efficacy, and combined interventions targeting the “risk hub” and multiple dominant pathways are required. The revised section adds combined intervention strategies for the pathway interaction network, clarifies the intervention principles and implementation methods, and provides real‐world multipathway combined intervention examples.

Combined intervention principles:

1. Target the “risk hub” first: Inhibit the activation of the chronic stress “risk hub” through psychological intervention and stress source management, reduce stress perception intensity, and cut off the signal input of the pathway interaction network.
2. Target dominant pathways: Identify the dominant pathological pathways of individual patients through comprehensive assessment (e.g., HPA axis dysfunction + ANS imbalance for hypertensive patients, inflammation + oxidative stress for diabetic patients), and implement pathway‐specific targeted interventions.
3. Combine multiple interventions: Integrate psychological intervention, lifestyle modification, dietary care, and exercise guidance to form a comprehensive combined intervention plan, and break the vicious cycle among pathways.
4. Dynamic adjustment: Conduct regular reassessment of patients, monitor the activation state of the “risk hub” and the improvement of dominant pathways, and dynamically adjust the intervention plan according to the assessment results.

Real‐world combined intervention example: A 60‐year‐old male patient with chronic stress, diagnosed with hypertension (150/100 mmHg) and mild depression, a comprehensive assessment shows dominant pathways: HPA axis dysfunction + ANS imbalance + mild chronic inflammation. Combined intervention plan: 1. risk hub intervention: 12 sessions of CBT + stress source management (retire early, reduce work pressure); 2. dominant pathway intervention: sleep care (melatonin supplementation) + Tai Chi exercise (40 min a day) + anti‐inflammatory diet plan; 3. dynamic monitoring: reassess physiological indicators and emotional state every 4 weeks. After 12 weeks of intervention, the patient’s blood pressure reduced to 130/80 mmHg, depression symptoms relieved, serum cortisol, HRV, and CRP all returned to normal ranges, and the pathway interaction network vicious cycle was broken.

## 7. Key Points and Prospects of Clinical Nursing Practice

### 7.1. Key Points of Clinical Nursing Practice

Based on the revision and improvement of the whole manuscript, the key points of clinical nursing practice for chronic stress‐related psychosomatic diseases are refined, with the core of “pathway‐based, precise nursing,” and the integration of biological mechanisms into the entire process of nursing assessment and intervention. The key points are summarized as five aspects [82–86]:

1. Establish a pathway‐based assessment system: Take the four core biological pathways and the “risk hub” model as the theoretical basis, combine physiological biomarkers and psychosocial assessment to achieve a comprehensive and precise assessment of chronic stress patients, and identify high‐risk populations and dominant pathological pathways.
2. Implement pathway‐targeted precise intervention: Develop personalized intervention plans according to the patient’s risk stratification and dominant pathways, strengthen the mechanistic link between nursing interventions and biological pathway modulation, avoid generalized and nonspecific intervention measures, and improve intervention efficacy.
3. Adopt an integrated biopsychosocial care approach: Integrate biological, psychological, and social factors into clinical nursing practice, pay attention to both the regulation of biological pathways and the improvement of psychological state and social support, and break the psychosomatic vicious cycle.
4. Strengthen multidisciplinary collaboration: Establish a multidisciplinary collaboration team including nursing, clinical medicine, psychology, nutrition, and rehabilitation, conduct joint assessment and intervention for patients with complex comorbidities, and provide comprehensive care services.
5. Emphasize long‐term follow‐up and self‐management: Conduct regular long‐term follow‐up for chronic stress patients, monitor the progression of the disease and the improvement of biological pathways; strengthen health education and self‐management training, guide patients to master stress coping skills and pathway‐targeted self‐care methods, and reduce disease recurrence.

### 7.2. Research and Practice Outlook

The revised section combines cutting‐edge research advances (peripheral stress response, stress–cancer crosstalk, photo‐neuro‐immuno‐endocrinology) and clinical nursing needs, and expands the research and practice prospects of chronic stress clinical nursing, with a focus on precision, personalization, and informatization, and four key research directions are proposed [87–89]:

1. Development of precise nursing assessment tools: Based on multiomics technology (genomics, metabolomics, microbiomics), explore specific biological markers of chronic stress for different psychosomatic diseases, develop noninvasive, rapid, and portable assessment tools (e.g., salivary biomarker detection kits, smart wearable devices for real‐time HRV, and cortisol monitoring), and improve the precision of nursing assessment.
2. Exploration of personalized pathway‐targeted interventions: Conduct large‐sample clinical trials to verify the efficacy of pathway‐targeted interventions for different populations (age, gender, disease type, genetic background); standardize the intervention methods, intensity, and duration; and develop personalized nursing intervention guidelines for chronic stress‐related psychosomatic diseases.
3. Application of informatization and intelligent nursing technology: Leverage mobile health (mHealth) technology (smart wristbands, mobile apps) to realize real‐time monitoring of patients’ stress levels, physiological indicators, and lifestyle habits; develop intelligent nursing decision‐making systems based on artificial intelligence (AI), which can automatically generate personalized intervention plans according to the patient’s assessment results and dynamically adjust them according to the follow‐up data.
4. Expansion of peripheral organ‐targeted nursing interventions: Based on the research progress of peripheral stress response systems (e.g., skin and gut), explore new peripheral organ‐targeted nursing intervention methods (e.g., skin phototherapy, gut microbiota intervention, and massage), and combine central and peripheral interventions to form a whole‐body stress response regulation system, improving the overall efficacy of nursing intervention.
5. Integration of chronic stress management into specialized nursing: Incorporate chronic stress management into the comprehensive nursing of various specialized diseases (e.g., cancer, cardiovascular disease, and diabetes), develop disease‐specific stress management nursing guidelines, and improve the comprehensive treatment effect and quality of life of patients with chronic diseases.

## 8. Conclusion

Chronic stress acts as a core risk hub for psychosomatic diseases, mediating the onset and progression of diverse multisystem psychosomatic diseases (including cardiovascular diseases, mental disorders, metabolic syndrome, and even cancer) through four core interconnected biological pathways: HPA axis dysfunction, ANS imbalance, abnormal inflammatory responses, and excessive oxidative stress. These pathways form a mutually reinforcing regulatory network, and the bidirectional interaction between the central neuroendocrine stress response system and peripheral stress response systems (e.g., skin neuro‐immuno‐endocrine system) further expands the scope of stress‐induced bodily damage, forming a whole‐body stress response network.

Clinical nursing, as a core component of chronic stress‐related disease management, must take biological pathways and the “risk hub” model as the theoretical basis, conduct comprehensive and precise assessment combining physiological biomarkers and psychosocial factors, identify high‐risk populations and dominant pathological pathways, and implement pathway‐targeted precise intervention—including psychological intervention to regulate HPA axis function, exercise/respiratory training to improve ANS imbalance, anti‐inflammatory diet to suppress chronic inflammation, and antioxidant supplementation to alleviate oxidative stress. Meanwhile, clinical nursing should integrate cutting‐edge research advances (melatonin application, moderate UV radiation, skin‐targeted intervention, stress–cancer crosstalk management), adopt an integrated biopsychosocial care approach, and strengthen multidisciplinary collaboration and long‐term follow‐up.

Future clinical nursing research and practice should focus on the development of precise assessment tools, the exploration of personalized pathway‐targeted interventions, the application of informatization and intelligent nursing technology, and the expansion of peripheral organ‐targeted interventions. By promoting the precision, personalization, and informatization of clinical nursing practice, we can provide higher‐quality nursing services for patients with chronic stress‐related psychosomatic diseases, effectively inhibit the activation of the chronic stress “risk hub,” break the vicious cycle among biological pathways, and ultimately achieve early prevention, effective intervention, and prognosis improvement of chronic stress‐related psychosomatic diseases.

## Funding

No funding was received for this manuscript.

## Conflicts of Interest

The authors declare no conflicts of interest.

## Data Availability

The data that support the findings of this study are available from the corresponding author upon reasonable request.
